# Transcriptional regulatory program in wild-type and retinoblastoma gene-deficient mouse embryonic fibroblasts during adipocyte differentiation

**DOI:** 10.1186/1756-0500-4-157

**Published:** 2011-05-26

**Authors:** Robab Hakim-Weber, Anne-M Krogsdam, Claus Jørgensen, Maria Fischer, Andreas Prokesch, Juliane G Bogner-Strauss, Stefan R Bornstein, Jacob B Hansen, Lise Madsen, Karsten Kristiansen, Zlatko Trajanoski, Hubert Hackl

**Affiliations:** 1 Department of Internal Medicine, Technical University Dresden, Germany; 2 Biocenter, Division of Bioinformatics, Innsbruck Medical University, Austria; 3 Cell Communication Team, Section of Cell and Molecular Biology, The Institute of Cancer Research, London, UK; 4 Institute for Genomics and Bioinformatics, Graz University of Technology, Austria; 5 Department of Biomedical Sciences, University of Copenhagen, Denmark; 6 Department of Biology, University of Copenhagen, Denmark; 7 National Institute of Nutrition and Seafood Research, Bergen, Norway

## Abstract

**Background:**

Although many molecular regulators of adipogenesis have been identified a comprehensive catalogue of components is still missing. Recent studies showed that the retinoblastoma protein (pRb) was expressed in the cell cycle and late cellular differentiation phase during adipogenesis. To investigate this dual role of pRb in the early and late stages of adipogenesis we used microarrays to perform a comprehensive systems-level analysis of the common transcriptional program of the classic 3T3-L1 preadipocyte cell line, wild-type mouse embryonic fibroblasts (MEFs), and retinoblastoma gene-deficient MEFs (Rb-/- MEFs).

**Findings:**

Comparative analysis of the expression profiles of 3T3-L1 cells and wild-type MEFs revealed genes involved specifically in early regulation of the adipocyte differentiation as well as secreted factors and signaling molecules regulating the later phase of differentiation. In an attempt to identify transcription factors regulating adipogenesis, bioinformatics analysis of the promoters of coordinately and highly expressed genes was performed. We were able to identify a number of high-confidence target genes for follow-up experimental studies. Additionally, combination of experimental data and computational analyses pinpointed a feedback-loop between Pparg and Foxo1.

To analyze the effects of the retinoblastoma protein at the transcriptional level we chose a perturbated system (Rb-/- MEFs) for comparison to the transcriptional program of wild-type MEFs. Gene ontology analysis of 64 deregulated genes showed that the Rb-/- MEF model exhibits a brown(-like) adipocyte phenotype. Additionally, the analysis results indicate a different or additional role for pRb family member involvement in the lineage commitment.

**Conclusion:**

In this study a number of commonly modulated genes during adipogenesis in 3T3-L1 cells and MEFs, potential transcriptional regulation mechanisms, and differentially regulated targets during adipocyte differentiation of Rb-/- MEFs could be identified. These data and the analysis provide a starting point for further experimental studies to identify target genes for pharmacological intervention and ultimately remodeling of white adipose tissue into brown adipose tissue.

## Introduction

Evidence accumulating during the past decades has convincingly revealed that adipocytes and adipose tissue are not only acting as a storage depot for fat, but are also actively involved in regulating whole body energy balance, and much attention has been dedicated to decipher molecular events and to identify factors involved in fat cell development. Although many molecular regulators have been identified, a comprehensive catalogue of components regulating adipogenesis is still missing. Therefore, a number of expression profiling studies using microarrays have been performed to monitor global gene expression profiles during *in vitro *adipocyte differentiation of several cell models [1].

Recently, we have studied global gene expression of 3T3-L1 cells during adipogenesis and identifying molecular mechanisms, biological processes, key enzymes, including many characterized and uncharacterized transcriptional regulators, thereby providing a gene expression atlas using functional annotation [2]. We showed that the mitotic clonal expansion phase plays an important role during adipocyte differentiation in the studied mouse cell model. One group of proteins - the pocket proteins including the retinoblastoma protein (pRb) and other members (p130, p107) - was expressed during both, the cell cycle phase and late cellular differentiation. This dual role of pRb in the early and late stages of adipogenesis was also described in other studies [3-6]. Pathways regulated by the retinoblastoma gene/protein might therefore be interesting novel targets in the combat to curb or to treat obesity and associated disorders.

Several studies on mechanisms and interactions have elucidated how pRb - the product of the Rb1 gene - fulfills its functions. pRb is phosphorylated by cyclin-dependent kinases thereby regulating E2F activity [5]. Other mechanisms include co-factor activity for none-E2F transcription factors, recruitment of chromatin remodeling complexes (including HDACs) and action as a non-chromatin associated protein adaptor [4,6,7]. The first evidence that pocket proteins are involved in adipocyte differentiation emerged by showing interactions with the C/EBP transcription factors [8,9]. It was shown that the ability of the C-terminally truncated large T antigen (TAg) to inhibit differentiation of white preadipocyte cell lines is dependent on the ability to sequester pRb family members [10]. Further studies showed that pRb and p107/p130 may have opposite effects on adipocyte differentiation [11,12].

Rb-/- mouse embryonic fibroblasts (MEFs) are unable to undergo adipose conversion using standard adipogenic inducers [8,13,14]. It was suggested that the underlying mechanism did not directly mirror cell cycle-related aspects of pRb function, as Rb-/- cells enter and exit mitotic clonal expansion with the same kinetics as wild-type cells [13]. Further it was shown that pRb is required for preadipocyte differentiation *in vivo *[12,15] and functions as a molecular switch determining white versus brown(-like) adipocyte differentiation [14,16]. Of note, adipose tissue-specific inactivation of Rb prevents [15] and haploinsufficiency of Rb attenuates diet induced obesity and insulin resistance [17]. pRb might also be involved in the lineage commitment to osteogenic or adipogenic differentiation of mesenchymal stem cells [18].

Thus, given the accumulated evidence of the involvement of pRb in adipogenesis we initiated a large scale gene expression study to elucidate its role during adipogenic differentiation. We first performed microarray gene expression analysis of wild-type mouse embryonic fibroblasts (MEFs) undergoing adipogenesis and compared these expression data with microarray gene expression data from 3T3-L1 cell adipogenesis to identify a transcriptional program common between the two cell models. Additionally, bioinformatics analyses were carried on the promoters of highly expressed genes to identify potential transcription factor binding sites and to generate hypotheses on transcription factor binding. To analyze the effects of the retinoblastoma protein at the transcriptional level we chose a perturbated system (Rb-/- MEFs) for comparison to the transcriptional program of wild-type MEFs.

## Results and Discussion

### Common transcriptional program of adipocyte differentiation in MEFs and 3T3-L1 cells

**Figure 1:**
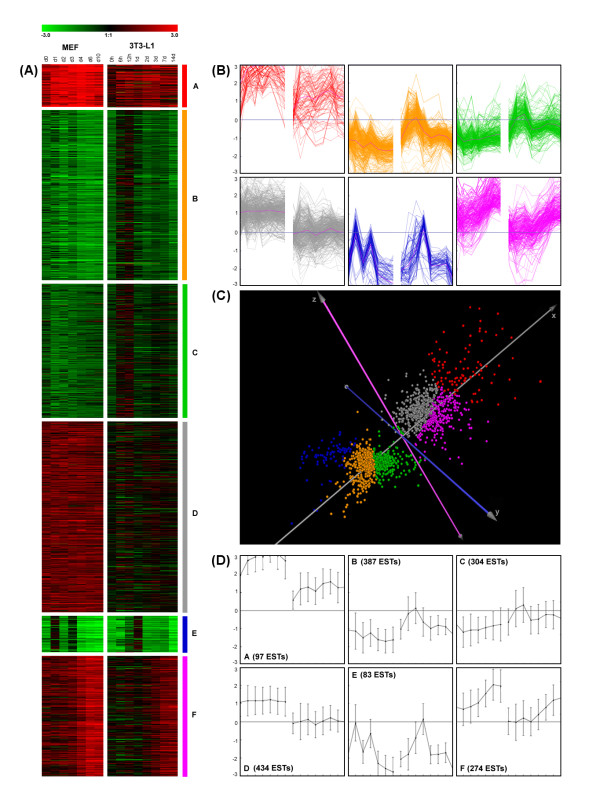
**Gene expression levels (log2-fold change) of genes from different time points during adipocyte differentiation in 3T3-L1 and MEF cells**. **(A) **Compact heatmaps from k-means clustering. **(B) **Line drawing centroid view (mean +/- SD). **(C) **Three-dimensional visualization of Principal Component Analysis (PCA) (color coding according k-means clustering) demonstrates cluster separation. **(D) **Line drawing for expression course of each gene individually.

To compare transcriptional regulation during adipocyte differentiation of the established 3T3-L1 cell line and mouse embryonic fibroblasts (MEFs), gene expression in MEFs was studied in three independent experiments with whole genome cDNA microarrays (>27k elements) using seven time points (d0, d1, d2, d3, d4, d6, d10) after hormonal induction in relation to expression levels at the preconfluent stage (similar to the generation of data from the previous 3T3-L1 study [2]) (Additional file 1). We validated the microarray data from MEFs by quantitative reverse transcription polymerase chain reaction (qPCR) analysis of the first differentiation experiment (see Figure S1 in Additional file 2) with six genes at several time points. Applying linear regression of the resulting 18 data points showed a coefficient of determination (*R*^*2*^) of 0.92 (Pearson correlation coefficient *r *= 0.96), which represented good agreement between microarray and qPCR results. Replicate results were also in good concordance with log2-fold changes averaged over the three independent differentiation experiments. Differentially expressed genes with similar combined expression profiles of the 3T3-L1 and MEF cells were grouped into 6 clusters by k-means clustering (Figure 1 and Additional file 1). Proficient quality of clustering was evident from different visualizations of the gene expression levels and separation of clusters using Principal Component Analysis (PCA) in three-dimensional view of the first three principal components and color-coding according to k-means clustering (Figure 1).

**Figure 2:**
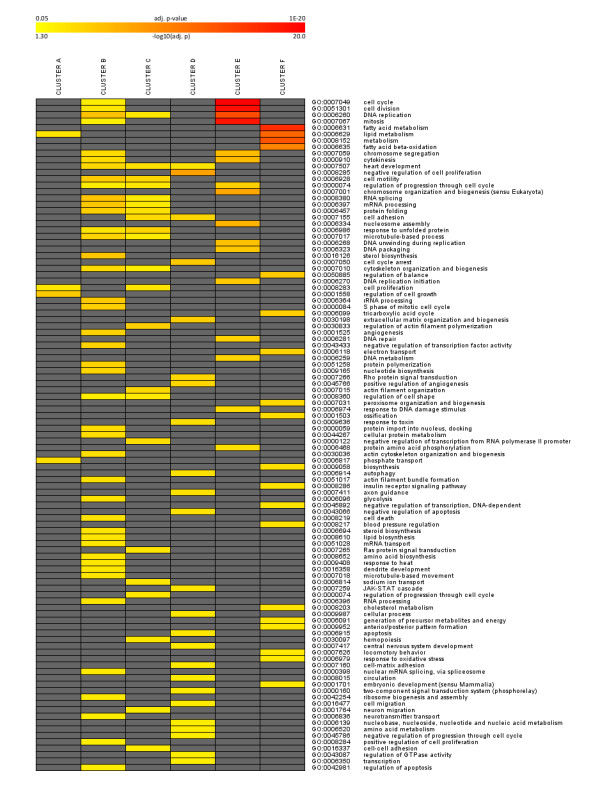
**Gene ontology analysis.** Heatmap for overrepresented gene ontology terms for genes in 6 clusters over several time points during adipocyte differentiation in 3T3-L1 and MEF cells as visualized in Figure 1 (color coding according to legend at the top, only gene ontology terms with adjusted *p *< 0.05 were considered) is shown.

Functional assignment of genes within each cluster by gene ontology analysis revealed a number of noticeable biological processes regulated at the transcriptional level (Figure 2). Most significantly overrepresented biological processes were 'cell cycle', 'cell division', 'DNA replication', and 'mitosis' indicating mainly the involvement of genes of cluster E and cluster B in cell cycle processes. Distinct peaks in the expression profiles of these genes at d1 and d3 showed that MEFs underwent two cycles of mitotic clonal expansion comparable to the 3T3-L1 cells showing - at the used sampling rate - one peak in the expression profiles at 24 h [2] which could reflect the first of two rounds of mitotic clonal expansions [19,20]. This observation is supported by reported phosphorylation events of the pRb in 3T3-L1 cells during differentiation [3,13] and the cyclin A2 (Ccna2) expression profile as shown in Additional file 1 and Additional file 2.

As evident from the gene ontology analysis of genes in cluster E, significantly overrepresented biological processes were 'fatty acid metabolism', 'lipid metabolism', 'metabolism', and 'fatty acid beta-oxidation'. Well known genes important for the development of the adipocyte phenotype were combined in cluster F with increasing expression over time, including transcription factors and nuclear receptors Pparg, Cebpa, Srebf1, Xbp1, Thra, Stat1, Nr0b2 (SHP), Nr1d1 (REV-ERB alpha), Nr3c1 (GR), Foxc1, Foxo1, Zhx1, and Zfp503. The recently identified gene coding for adipocyte plasma membrane-associated protein [21] was also shown to be highly expressed at late stages of adipogenesis of MEFs (cluster F).

Highly expressed genes over the differentiation course, especially at the middle stage of adipogenesis (after mitotic clonal expansion) could be found in cluster A. In this group we observed not only transcription factors like Klf9 [22], and Foxo3a [23] but also secreted factors (as studied in human [24]) including Apod [25], Ccdc80 [26], Adamts5 [27], Mmp2 [28], and Ptn [29], as well as enzymes, signaling molecules, and other factors such as Xdh [30], Enpp2 [31], Rgs2 [32], Txnip [33], Tsc22d3[34], H6pd [35], Igfbp2 [36], and Igfbp3 [37]. Hence, the genes in cluster A represent potential novel players in adipogenesis and are therefore candidates to be studied in more detail experimentally.

To identify candidates selectively associated with the MEF cell model, genes upregulated during MEF differentiation which showed marginal changes in 3T3-L1 adipocytes compared to the preconfluent stage of 3T3-L1 are of major interest (cluster D). Genes in this cluster were overrepresented in the GO terms 'heart development', 'negative regulation of cell proliferation', 'cell adhesion', 'extra cellular matrix organization and biogenesis', and 'positive regulation of angiogenesis'. The expressed transcription factors include Arnt, Arnt2, Atf5, Ets2, Egr1, Fosl2, JunB, Nfia, Prdm4, Stat2, Stat3, Tef, and Tcf20 which might be involved specifically in early regulation of adipocyte differentiation in the primary cell model as opposed to the already committed 3T3-L1 preadipocytes.

### Transcription factors regulating gene expression during adipocyte differentiation

The terminal development of a fat cell is driven by a transcriptional cascade governed mainly by Pparg and Cebpa (as reviewed in [38,39]). The transcriptional events directing early adipogenic processes, concerted by molecular events prior to the activation of Pparg and Cebpa have not been exhaustively studied. Hence, we asked if there are common regulators of expression of genes within each cluster, especially those with expression profiles similar to a transcription factor and its target genes. To address this question, we calculated overrepresentation of sequence motifs (response elements) within a genomic region (including promoter) from -3 kb to +2 kb relative to transcription start site (TSS) of those genes (Additional file 3).

**Figure 3:**
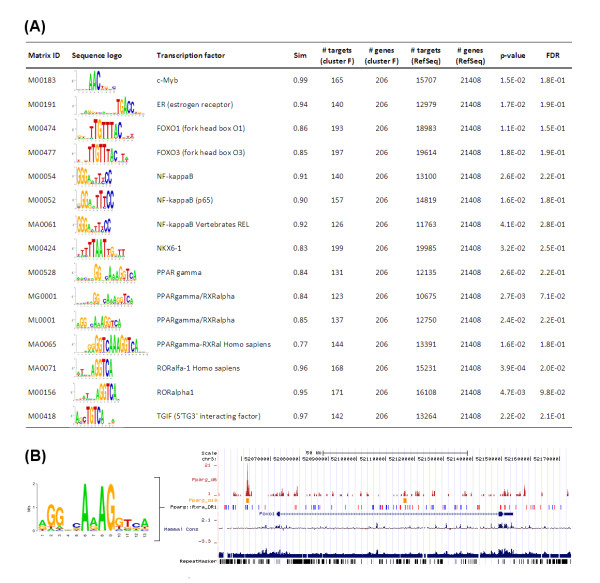
***In silico* promoter analysis and genomic organization**. **(A) **Statistically overrepresented (*p *< 0.05) potential transcription factor binding sites (TFBS) identified by *in silico *analysis of genomic regions from -3 kb to + 2 kb around transcription start sites of genes with increasing gene expression at late stages of adipocyte differentiation (cluster F) using position frequency (weight) matrices from TRANSFAC and JASPAR. Corresponding sequence logos are shown and number of targets within the dataset (cluster) is given and tested against the number of targets from all RefSeq transcripts using Fishers exact test. Note only transcription factor motifs are considered which are exclusively overrepresented in cluster F. **(B) **The genomic organization around Foxo1 using the UCSC genome browser (NCBI37/mm9) with high similarity to the Pparg-Rxra DR-1 motif (similarity score > 0.90) (blue '+' strand, red '-'strand), Pparg binding regions as identified by previous studies at day 6 [42] (dark red) and day 10 [43] (orange) of 3T3-L1 adipocyte differentiation.

From the transcription factors with exclusively overrepresented binding sites (Figure 3A) in promoters of genes from cluster F (i.e. genes with increasing expression towards terminal adipocyte differentiation) only Foxo1 and the key player in adipogenesis Pparg have expression profiles in cluster F. It has been previously shown that Foxo1 can interact with Pparg [40]. Furthermore, Foxo1 can bind to the Pparg promoter region and suppress Pparg expression [41]. Our analyses suggest that a feed-back loop from Pparg to Foxo1 might exist. To provide further evidence we analyzed two datasets of DNA binding by Pparg identified by chromatin immunoprecipitation (ChIP) followed by sequencing [42] or by microarray analysis [43] during 3T3-L1 adipocyte differentiation. Integrative analysis of these datasets and Pparg binding sites in genomic regions around genes with expression profile similar to that of Pparg in both the 3T3-L1 cells and MEFs (cluster F), revealed that Foxo1 might indeed be regulated by Pparg as indicated in Figure 3B. Thus, our data, experimental results from other studies, and further computational analysis pinpointed a feed-back loop between Pparg and Foxo1.

We have recently identified 2310001A20Rik (Apmap), as well as the retrotransposed genes 6530401D17Rik (Arxes1) and 2900062L11Rik (Arxes2) as Pparg targets and that they are required for adipogenesis [21,44]. A number of other genes annotated as Riken clones (like 5730469M10Rik and F630110N24Rik) included in cluster F show noticeable binding by Pparg in 3T3-L1 cells in the first intron and hence might be interesting targets in follow-up experimental studies. Additional target genes were Acadm, Adcy5, Dgat1, Etfb, Ech1, Hmox1, Mdh1, Mgst3, Pparg, Pla2g15, Phldb1, Sorbs1, and Srebf1 as indicated in both chromatin immunoprecipitation (ChIP) studies [42,43] of Pparg binding at the same genomic region near TSS (< 1 kb) or in an intron (peak height > 70) (see additional website http://icbi.at/adipo). There was also supporting evidence of overrepresented known cell cycle related transcription factor binding sites in the genes of cluster B, C, and E (including NF-Y, E2F, cMyc:Max, USF, c-ETS). In cluster A exclusively overrepresented transcription factor binding sites includes STAT3, STAT5A, STAT5B, MEF-2, Hand1:E47, and NF-1. The genes encoding for STAT3 and NF-I could also be found in this cluster, indicating that these two transcription factors share similar expression profiles as their target genes in cluster A.

### Transcriptional program of Rb-/- MEFs

**Figure 4:**
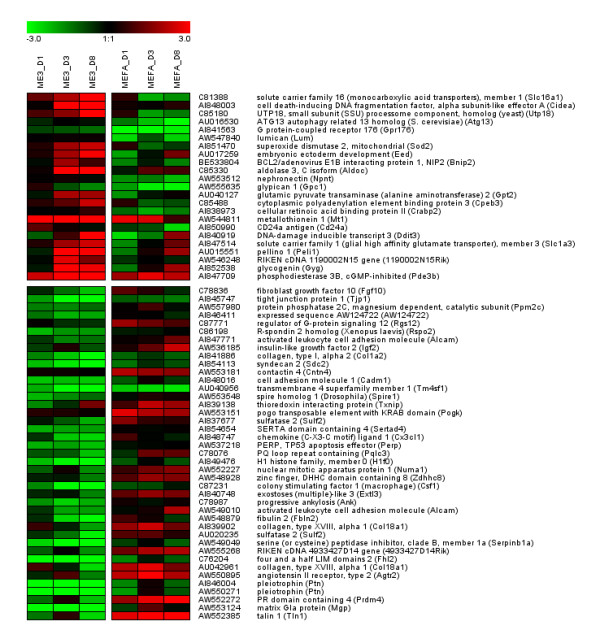
**Gene expression profiles in Rb-/- MEFs. **Gene expression levels (log2-fold change) shown as heatmap for samples from 3 different time points (d1, d3, d8) related to d0 immediately before hormonal induction of Rb-/- MEF (ME3) and Rb+/+ MEF (MEFA) differentiation. Only genes with sum of differences > 5 or sum of differences < -5 are shown.

Recent studies showed that the retinoblastoma protein (pRb) was expressed in the cell cycle and late cellular differentiation phase during adipogenesis. The interesting Rb1 gene expression profiles during 3T3-L1 and wild-type MEF adipogenesis are also shown in Additional file1. Consequently, we studied the effect of perturbation of the MEF adipogenesis system by lack of Rb1 by a direct comparison of the global expression levels of Rb-/- MEF (ME3) cells to those of the wild-type MEFs (MEFA) at 3 time points (d1, d3, d8) during adipocyte differentiation in relation to the expression at day 0, immediately before hormonal induction using again whole genome cDNA microarrays (> 27k elements). Since Rb-/- MEFs do not differentiate into adipocytes in response to a standard adipogenic cocktail, the proadipogenic Pparg agonist Rosiglitazone was added in both the Rb-/- MEF (ME3) and the Rb+/+ MEF (MEFA) differentiation experiments. We calculated several distance measures (sum of differences, Manhattan distance, Euclidean distance, and Pearson correlation) between the expression profiles of ME3 and MEFA cells for 3118 differentially expressed ESTs (Additional file 4) and focused on 64 upregulated genes (sum of differences > 4) and 90 downregulated genes (sum of differences < -4). For clarity, gene expression levels of only the most deregulated ESTs were visualized as heatmap in Figure 4 (23 ESTs with sum of differences > 5 and 41 ESTs with sum of differences < -5).

**Figure 5:**
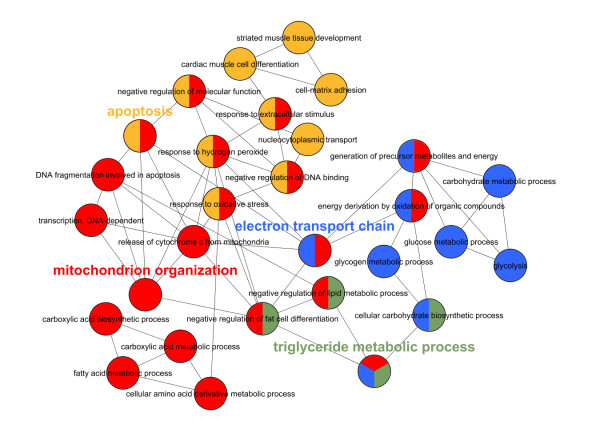
**ClueGO analysis **[57]**of 64 genes upregulated during Rb-/- MEF adipocyte differentiation in comparison to Rb+/+ MEF adipocyte differentiation (sum of differences of log2-fold change > 4)**. Nodes (circles) represent gene ontology terms. Connections between two nodes (edges) indicate that two gene ontology terms share genes from the considered dataset (agreement measure kappa ≥ 0.3). The calculated kappa score is also used for defining functional groups, which are indicated by the same color. The most prominent gene ontology term for each group (theme) is highlighted: *mitochondrion organization *(red), *electron transport *(blue), *triglyceride metabolic process *(green), *apoptosis *(orange).

As evident from previous studies, microarray and qPCR analysis revealed that Ucp1, Ppargc1a, Prdm16, and Cidea are highly upregulated in Rb-/- MEF (ME3) adipocyte differentiation compared to Rb+/+ MEF (MEFA) adipocyte differentiation (see [14,16,45], Additional file 2, and Additional file 4). Gene ontology analysis from the 64 deregulated genes corroborated the brown(-like) adipocyte phenotype of the Rb-/- MEF model: the most significant overrepresented gene ontology term was 'generation of precursor metabolites and energy' (FDR = 3.1E-05) (including electron transferring flavoprotein, alpha polypeptide (Etfa) and NADH dehydrogenase (ubiquinone) Fe-S protein 1 (Ndufs1) also involved in oxidative phosphorylation a prominent process in brown adipose tissue). Analysis with the tool ClueGO (Figure 5) showed prominent gene ontology terms 'mitochondrion organization', 'electron transport', 'triglyceride metabolic processes', and 'apoptosis'. These are related to hallmarks of a brown adipocyte phenotype such as mitochondrial biogenesis, metabolic processes, electron transport, oxidative phosphorylation, and uncoupling. Many of the activated genes are encoding for enzymes involved in glucose or lipid metabolism and one gene for a transcription factor (Myc).

It is noteworthy that gene set enrichment analyses of the ranked gene list with gene sets from brown adipose tissue versus white adipose tissue [46] and brown and white preadipocytes at the differentiating stages [47] showed a high enrichment score at least to the first gene set supporting the notion that Rb is involved in the switch between the white and brown adipocyte differentiation process but also indicated expression phenotypic differences between brown adipocytes and differentiated Rb-/- MEFs (Additional file 5). These results suggest a different and/or additional role of pRb. We have seen that Rb-/- MEFs behave more like primary cells from inguinal white adipose tissue than classic brown adipose tissue [48]. It was also shown that pRb could have a different role as it might be involved in the lineage commitment to either osteogenic or adipogenic differentiation of mesenchymal stem cells [18].

## Conclusion

In this work we studied adipocyte differentiation of wild-type MEFs and Rb-/- MEFs into mature adipocytes using microarrays and comprehensive bioinformatics analyses. We identified novel candidates involved in early adipogenesis as well as high-confidence genes targeted by Pparg. Our findings corroborate the brown-like phenotype of the Rb-/- MEFs. Based on the analyses we also propose the existence of a feed-back loop between Pparg and Foxo1 adding a novel facet to the regulatory network orchestrating adipocyte differentiation.

The data and the analysis provide a starting point for further experimental studies to identify target genes for pharmacological intervention and ultimately remodeling of white adipose tissue into brown adipose tissue.

## Materials and methods

### Adipocyte differentiation of wild-type (Rb+/+) and Rb-/- mouse embryonic fibroblasts

Generation of Rb-/-MEFs and wild-type MEFs were previously performed at the Division of Cancer Biology, Danish Cancer Society, Copenhagen, DK and described in [49]; detailed information on the origin of these cells and disruption of the Rb-1 gene in mice can be found in [50]. A batch of the Rb-/- and Rb+/+ MEFs were kindly provided by Jiri Lukas as already used in other studies (e.g. [14]). No animals or human subjects were directly involved in the current study that would make ethical approval and consent required. Wild-type mouse embryo fibroblasts (MEFs) were differentiated at the Department of Biochemistry and Molecular Biology, University of Southern Denmark, Odense, DK according to the protocol described in [13]. Briefly, cells were induced to differentiation by the MDI protocol (1 μM dexamethasone (DEX), 0.5 mM isobutyl methylxanthine (MIX), and 5 μg/ml insulin). DEX and MIX were omitted after day 2, but insulin was added throughout the differentiation. Cells were passaged and differentiated in AmnioMax (Gibco) supplemented with 7,5% fetal bovine serum. Nutrition media were changed every second day. Rb-/- MEFs and wild-type MEFs (Rb+/+) for comparison were differentiated using the identical protocol, but adding Pparg ligand BRL49653 (0.5 μM) to the induction cocktail throughout the whole differentiation process. All differentiation experiments were performed in triplicate. Cells were harvested and total RNA was isolated at the preconfluent stage and at seven time points after induction (0, 1, 2, 3, 4, 6, and 10 days) with TRIzol reagent (Invitrogen). For the Rb-/- and Rb+/+ MEF differentiation, cells were harvested immediately before (reference) and at three time points (1, 3, and 8 days) after induction. For each independent experiment RNA was pooled from three different culture dishes for each time point and from twenty dishes for each reference. The quality of the RNA was checked using Agilent 2100 Bioanalyzer RNA assays (Agilent Technologies) by inspection of the 28S and 18S ribosomal RNA intensity peaks.

### Gene expression and data analysis

The mouse cDNA microarrays and the hybridization protocols used have been described previously [2]. Briefly, the spotted microarrays contain 27,000 elements with mouse cDNA clones representing 16,000 different genes (UniGene clusters). 10-25 micrograms (dependent on replicated experiment) of total RNA from each time point was reverse transcribed into cDNA, which was then indirectly labeled with Cy5 and Cy3, respectively. To account for technical variation, procedures were repeated using the same samples with reversed dye assignment. The microarrays were pre-hybridized with 5× SSC, 0.1% SDS,1% BSA. Pair-wise labeled cDNA samples were combined and 20 μg of mouse Cot1 DNA and 20 μg of poly(A) DNA were added. The mixture was hybridized onto the slides overnight at 42°C. Following washing, slides were scanned with a GenePix 4000B microarray scanner (Axon Instruments) at 10 μm resolution. The resulting TIFF images were analyzed with GenePix Pro 4.1 software (Axon Instruments). Features were filtered for low-quality spots. Following subtraction of the local background, the arrays were global median and dye-swap normalized using ArrayNorm [51] and the resulting ratios log2 transformed. All experimental parameters, raw data, and transformed data were uploaded to the microarray database MARS [52] and submitted via MAGE-ML export to a public repository (ArrayExpress [53], Accession Nos. E-MARS-12/E-MARS-13 and A-MARS-7). After outliers were removed the median expression values of replicated ESTs on the microarray were calculated. Only ESTs with *z *> 1.5 and significant replication (*p *< 0.05) in at least one time point using the z test and implying standard normal distribution were considered differentially expressed and used for further analysis [54,55]. Expression values and z scores for differentially expressed ESTs were averaged over biological replicates and annotated using the RefSeq database (MegaBLAST E-value < 1E-30) and Entrez Gene database. Expression profiles for MEF data and 3T3-L1 data [2] were combined based on the same EST ID and profiles were grouped into 6 clusters by k-means clustering and validated by principal component analysis. Gene ontology term overrepresentation analysis for the different gene sets (clusters) were performed using Fisher exact test and p-values were adjusted for multiple hypothesis tests based on the Benjamini-Hochberg method (http://genome.tugraz.at/ORA). Several distance measures between the expression profiles of the Rb-/- MEF (ME3) and Rb+/+ MEF (MEFA) differentiation models (sum of differences, Manhattan distance, Euclidean distance, Pearson correlation coefficient) were calculated. Over-represented gene ontology (GO) terms for up- (sum of differences > 4) or downregulated (sum of differences < -4) genes were identified using DAVID [56] based on gene symbols. Up-regulated genes were functionally grouped into gene ontology networks using the Cytoscape plug-in ClueGO [57]. All calculations were implemented in PERL 5.8.0 or R 2.10.1, cluster analyses and visualizations were performed using Genesis [58]. Gene set enrichment analysis (GSEA) [59] was performed using a tool from http://www.broadinstitute.org/gsea.

### qPCR analysis

Microarray expression results were confirmed with qPCR. cDNA was synthesized from 1.25 μg total RNA in 20 μl using random hexamers and SuperScript II reverse transcriptase (Invitrogen). The design of LUX™ primers was done using the Invitrogen web service. Quantitative RT-PCR analyses were performed starting with 12 ng reverse transcribed total RNA, with 0.5× Platinum Quantitative PCR SuperMix-UDG (Invitrogen), with a ROX reference dye, and with a 200 nM concentration of both LUX™ labeled sense and antisense primers (Invitrogen) in a 25 μl reaction. Ribosomal 18S RNA amplifications were used to account for variability in the initial quantities of cDNA. The relative quantification with respect to the calibrator (preconfluent stage) was determined using the ΔΔCt method and compared with the normalized ratios resulting from microarray experiments. To better characterize Rb-/- MEF (ME3) and Rb+/+ MEF (MEFA) cells, triplicate-qPCR analyses were performed with 4.5 ng cDNA, 200 nM primer, and Platinum SYBR green mastermix (Invitrogen). Data were analyzed using a real-time PCR management and analysis system [60]. Values were normalized to Uxt, Ct values and PCR efficiencies were derived as in [61]. All qPCR analyses were performed on an ABI PRISM 7000 detection system (Applied Biosystems). Primer sequences can be found in Additional file 2.

### In silico analysis of transcription factor binding sites

Statistically overrepresented (*p *< 0.05) potential transcription factor binding sites (TFBS) were identified by *in silico *analysis of genomic regions from -3 kb to + 2 kb around transcription start sites of genes within each cluster and all RefSeq transcripts using known (TRANSFAC [62], JASPAR [63]), and newly compiled position frequency (weight) matrices (http://genome.tugraz.at/Logo). Potential binding sites were identified if the similarity score for each PWM was equal or above the threshold (allowing 1 binding site with 10kb of mouse coding sequences) based on an implementation of the MatInspector algorithm [64]. The number of targets (showing at least one TFBS) within the gene set (cluster) was tested against the number of targets from all RefSeq transcripts using Fishers exact test and p-value was adjusted for multiple hypothesis testing based on the false discovery rate according to the method of Benjamini-Hochberg [65]. Genome organization around genes (genomic region from 20 kb upstream of transcription start to 20 kb downstream of transcription end) showing similar expression profile as Pparg were constructed using UCSC genome browser (NCBI37/mm9), custom track data from chromatin immune precipitation followed by sequencing at day 6 [42] or microarray analysis at day 10 [43] during 3T3-L1 adipocyte differentiation, and Pparg-Rxra direct repeats 1 (DR1) motifs (similarity score > 0.90) (see additional website http://icbi.at/adipo).

## Competing interests

The authors declare that they have no competing interests.

## Authors' contributions

Performed and conceived MEF and Rb-/- MEF cell culture experiment: CJ, JBH, LM, KK. Oversee qPCR analysis: AMK, HH. Generation of microarray slides: AP. Microarray experiments: HH. Bioinformatics and statistical analysis: MF, HH. Data analysis and interpretation: RHW, AMK, AP, JBS, SRB, ZT, HH. Wrote the paper: RHW, ZT, HH. All authors read and approved the manuscript.

## Supplementary Material

Additional file 1 **Gene expression levels (log2-fold change) of 1579 in 6 cluster grouped ESTs at several time points during adipocyte differentiation of MEFs and 3T3-L1 cells (as visualized in Figure **3**)**. ESTs were annotated according to the mouse RefSeq database (NCBI MegaBLAST E-value < 1E-30) and Entrez Gene database. Unique genes within each cluster were identified and overrepresented gene ontology (GO) terms are provided. Click here for file

Additional file 2 **Results and primer sequences from q-RT-PCR analysis for validation of microarray experiments and to identify expression levels for specific genes during ME3 and MEFA differentiation**. Click here for file

Additional file 3 **Statistically overrepresented (*p *< 0.05) potential transcription factor binding sites (TFBS) identified by *in silico *analysis of genomic regions from -3 kb to + 2 kb around transcription start sites of genes with increasing gene expression at late stages of adipocyte differentiation (cluster F) using position frequency (weight) matrices from TRANSFAC and JASPAR**. Corresponding sequence logos from http://genome.tugraz.at/Logo are shown and number of targets within the dataset (cluster) is given and tested against the number of targets from all RefSeq transcripts using Fishers exact test. Note only transcription factor motifs are considered which are exclusively overrepresented in cluster F. Click here for file

Additional file 4 **Gene expression levels (log2-fold change) of 3118 ESTs for samples from 3 different time points (d1, d3, d8) related to d0 immediately before hormonal induction of Rb-/- MEF (ME3) and Rb+/+ MEF (MEFA) differentiation. **Several distance measures between the expression profiles of the two cell differentiation models (sum of differences, Manhattan distance, Euclidean distance, Pearson correlation coefficient) are provided. Unique genes upregulated (sum of differences > 4) and downregulated (sum of differences < -4) in the ME3 cell differentiation model compared to the MEFA differentiation are emphasized and overrepresented gene ontology (GO) terms are provided. Click here for file

Additional file 5 **Gene set enrichment analysis of the gene list from comparison of Rb-/- MEFs and Rb+/+ MEFs during adipocyte differentiation ranked by sum of differences in a gene set of significantly upregulated (log2-fold change > 2) genes in brown adipose tissue versus white adipose tissue **[46]**and in a gene set with significantly upregulated (log2-fold change > 1) genes in brown versus white preadipocytes at the differentiating stages **[47]. Click here for file

## References

[B1] Prokesch A Hackl H Hakim-Weber R Bornstein SR Trajanoski Z Novel insights into adipogenesis from omics data Curr Med Chem 2009 16 23 2952 2964 10.2174/092986709788803132 19689276 PMC2765082

[B2] Hackl H Burkard TR Sturn A Rubio R Schleiffer A Tian S Quackenbush J Eisenhaber F Trajanoski Z Molecular processes during fat cell development revealed by gene expression profiling and functional annotation Genome Biol 2005 6 13 R108 10.1186/gb-2005-6-13-r108 16420668 PMC1414107

[B3] Hansen JB te RH Kristiansen K Novel function of the retinoblastoma protein in fat: regulation of white versus brown adipocyte differentiation Cell Cycle 2004 3 6 774 778 15197340

[B4] Burkhart DL Sage J Cellular mechanisms of tumour suppression by the retinoblastoma gene Nat Rev Cancer 2008 8 9 671 682 10.1038/nrc2399 18650841 PMC6996492

[B5] Fajas L Landsberg RL Huss-Garcia Y Sardet C Lees JA Auwerx J E2Fs regulate adipocyte differentiation Dev Cell 2002 3 1 39 49 10.1016/S1534-5807(02)00190-9 12110166

[B6] Morris EJ Dyson NJ Retinoblastoma protein partners Adv Cancer Res 2001 82 1 54 11447760 10.1016/s0065-230x(01)82001-7

[B7] Fajas L Egler V Reiter R Hansen J Kristiansen K Debril MB Miard S Auwerx J The retinoblastoma-histone deacetylase 3 complex inhibits PPARgamma and adipocyte differentiation Dev Cell 2002 3 6 903 910 10.1016/S1534-5807(02)00360-X 12479814

[B8] Chen PL Riley DJ Chen Y Lee WH Retinoblastoma protein positively regulates terminal adipocyte differentiation through direct interaction with C/EBPs Genes Dev 1996 10 21 2794 2804 10.1101/gad.10.21.2794 8946919

[B9] Cole KA Harmon AW Harp JB Patel YM Rb regulates C/EBPbeta-DNA-binding activity during 3T3-L1 adipogenesis Am J Physiol Cell Physiol 2004 286 2 C349 C354 10.1152/ajpcell.00255.2003 14576085

[B10] Higgins C Chatterjee S Cherington V The block of adipocyte differentiation by a C-terminally truncated, but not by full-length, simian virus 40 large tumor antigen is dependent on an intact retinoblastoma susceptibility protein family binding domain J Virol 1996 70 2 745 752 8551611 10.1128/jvi.70.2.745-752.1996 PMC189875

[B11] Classon M Kennedy BK Mulloy R Harlow E Opposing roles of pRB and p107 in adipocyte differentiation Proc Natl Acad Sci USA 2000 97 20 10826 10831 10995476 10.1073/pnas.190343597 PMC27108

[B12] Scime A Grenier G Huh MS Gillespie MA Bevilacqua L Harper ME Rudnicki MA Rb and p107 regulate preadipocyte differentiation into white versus brown fat through repression of PGC-1alpha Cell Metab 2005 2 5 283 295 10.1016/j.cmet.2005.10.002 16271529

[B13] Hansen JB Petersen RK Larsen BM Bartkova J Alsner J Kristiansen K Activation of peroxisome proliferator-activated receptor gamma bypasses the function of the retinoblastoma protein in adipocyte differentiation J Biol Chem 1999 274 4 2386 2393 10.1074/jbc.274.4.2386 9891007

[B14] Hansen JB Jorgensen C Petersen RK Hallenborg P De Matteis R Boye HA Petrovic N Enerback S Nedergaard J Cinti S Retinoblastoma protein functions as a molecular switch determining white versus brown adipocyte differentiation Proc Natl Acad Sci USA 2004 101 12 4112 4117 10.1073/pnas.0301964101 15024128 PMC384703

[B15] Dali-Youcef N Mataki C Coste A Messaddeq N Giroud S Blanc S Koehl C Champy MF Chambon P Fajas L Adipose tissue-specific inactivation of the retinoblastoma protein protects against diabesity because of increased energy expenditure Proc Natl Acad Sci USA 2007 104 25 10703 10708 10.1073/pnas.0611568104 17556545 PMC1965576

[B16] Hallenborg P Feddersen S Madsen L Kristiansen K The tumor suppressors pRB and p53 as regulators of adipocyte differentiation and function Expert Opin Ther Targets 2009 13 2 235 246 10.1517/14712590802680141 19236241

[B17] Mercader J Ribot J Murano I Feddersen S Cinti S Madsen L Kristiansen K Bonet ML Palou A Haploinsufficiency of the retinoblastoma protein gene reduces diet-induced obesity, insulin resistance, and hepatosteatosis in mice Am J Physiol Endocrinol Metab 2009 297 1 E184 193 10.1152/ajpendo.00163.2009 19417128

[B18] Calo E Quintero-Estades JA Danielian PS Nedelcu S Berman SD Lees JA Rb regulates fate choice and lineage commitment in vivo Nature 2010 466 7310 1110 1114 10.1038/nature09264 20686481 PMC2933655

[B19] Tang QQ Otto TC Lane MD Mitotic clonal expansion: a synchronous process required for adipogenesis Proc Natl Acad Sci USA 2003 100 1 44 49 10.1073/pnas.0137044100 12502791 PMC140878

[B20] Morrison RF Farmer SR Role of PPARgamma in regulating a cascade expression of cyclin-dependent kinase inhibitors, p18(INK4c) and p21(Waf1/Cip1), during adipogenesis J Biol Chem 1999 274 24 17088 17097 10.1074/jbc.274.24.17088 10358062

[B21] Bogner-Strauss JG Prokesch A Sanchez-Cabo F Rieder D Hackl H Duszka K Krogsdam A Di Camillo B Walenta E Klatzer A Reconstruction of gene association network reveals a transmembrane protein required for adipogenesis and targeted by PPARgamma Cell Mol Life Sci 2010 67 23 4049 4064 10.1007/s00018-010-0424-5 20552250 PMC11115701

[B22] Pei H Yao Y Yang Y Liao K Wu JR Kruppel-like factor KLF9 regulates PPARgamma transactivation at the middle stage of adipogenesis Cell Death Differ 2011 18 2 315 327 10.1038/cdd.2010.100 20725087 PMC3131894

[B23] Luo W Cao J Li J He W Adipose tissue-specific PPARgamma deficiency increases resistance to oxidative stress Exp Gerontol 2008 43 3 154 163 10.1016/j.exger.2007.11.002 18083318

[B24] Zhong J Krawczyk SA Chaerkady R Huang H Goel R Bader JS Wong GW Corkey BE Pandey A Temporal profiling of the secretome during adipogenesis in humans J Proteome Res 2010 9 10 5228 5238 10.1021/pr100521c 20707391 PMC2948433

[B25] Hummasti S Laffitte BA Watson MA Galardi C Chao LC Ramamurthy L Moore JT Tontonoz P Liver X receptors are regulators of adipocyte gene expression but not differentiation: identification of apoD as a direct target J Lipid Res 2004 45 4 616 625 10.1194/jlr.M300312-JLR200 14703507

[B26] Tremblay F Revett T Huard C Zhang Y Tobin JF Martinez RV Gimeno RE Bidirectional modulation of adipogenesis by the secreted protein Ccdc80/DRO1/URB J Biol Chem 2009 284 12 8136 8147 10.1074/jbc.M809535200 19141617 PMC2658107

[B27] Voros G Sandy JD Collen D Lijnen HR Expression of aggrecan(ases) during murine preadipocyte differentiation and adipose tissue development Biochim Biophys Acta 2006 1760 12 1837 1844 17011710 10.1016/j.bbagen.2006.08.016

[B28] Dubois SG Tchoukalova YD Heilbronn LK Albu JB Kelley DE Smith SR Fang X Ravussin E Potential role of increased matrix metalloproteinase-2 (MMP2) transcription in impaired adipogenesis in type 2 diabetes mellitus Biochem Biophys Res Commun 2008 367 4 725 728 10.1016/j.bbrc.2007.12.180 18182154 PMC2747303

[B29] Gu D Yu B Zhao C Ye W Lv Q Hua Z Ma J Zhang Y The effect of pleiotrophin signaling on adipogenesis FEBS Lett 2007 581 3 382 388 10.1016/j.febslet.2006.12.043 17239862

[B30] Cheung KJ Tzameli I Pissios P Rovira I Gavrilova O Ohtsubo T Chen Z Finkel T Flier JS Friedman JM Xanthine oxidoreductase is a regulator of adipogenesis and PPARgamma activity Cell Metab 2007 5 2 115 128 10.1016/j.cmet.2007.01.005 17276354

[B31] Simon MF Daviaud D Pradere JP Gres S Guigne C Wabitsch M Chun J Valet P Saulnier-Blache JS Lysophosphatidic acid inhibits adipocyte differentiation via lysophosphatidic acid 1 receptor-dependent down-regulation of peroxisome proliferator-activated receptor gamma2 J Biol Chem 2005 280 15 14656 14662 10.1074/jbc.M412585200 15710620

[B32] Nishizuka M Honda K Tsuchiya T Nishihara T Imagawa M RGS2 promotes adipocyte differentiation in the presence of ligand for peroxisome proliferator-activated receptor gamma J Biol Chem 2001 276 32 29625 29627 10.1074/jbc.C100272200 11418611

[B33] Chutkow WA Birkenfeld AL Brown JD Lee HY Frederick DW Yoshioka J Patwari P Kursawe R Cushman SW Plutzky J Deletion of the alpha-arrestin protein Txnip in mice promotes adiposity and adipogenesis while preserving insulin sensitivity Diabetes 2010 59 6 1424 1434 10.2337/db09-1212 20299477 PMC2874703

[B34] Lee MJ Gong DW Burkey BF Fried SK Pathways regulated by glucocorticoids in omental and subcutaneous human adipose tissues: a microarray study Am J Physiol Endocrinol Metab 2011 300 3 E571 580 10.1152/ajpendo.00231.2010 21189358 PMC3279304

[B35] Senesi S Marcolongo P Manini I Fulceri R Sorrentino V Csala M Banhegyi G Benedetti A Constant expression of hexose-6-phosphate dehydrogenase during differentiation of human adipose-derived mesenchymal stem cells J Mol Endocrinol 2008 41 3 125 133 10.1677/JME-08-0028 18586838

[B36] Wheatcroft SB Kearney MT Shah AM Ezzat VA Miell JR Modo M Williams SC Cawthorn WP Medina-Gomez G Vidal-Puig A IGF-binding protein-2 protects against the development of obesity and insulin resistance Diabetes 2007 56 2 285 294 10.2337/db06-0436 17259371 PMC4295171

[B37] Chan SS Schedlich LJ Twigg SM Baxter RC Inhibition of adipocyte differentiation by insulin-like growth factor-binding protein-3 Am J Physiol Endocrinol Metab 2009 296 4 E654 663 10.1152/ajpendo.90846.2008 19141684

[B38] Farmer SR Transcriptional control of adipocyte formation Cell Metab 2006 4 4 263 273 10.1016/j.cmet.2006.07.001 17011499 PMC1958996

[B39] Rosen ED MacDougald OA Adipocyte differentiation from the inside out Nat Rev Mol Cell Biol 2006 7 12 885 896 10.1038/nrm2066 17139329

[B40] Dowell P Otto TC Adi S Lane MD Convergence of peroxisome proliferator-activated receptor gamma and Foxo1 signaling pathways J Biol Chem 2003 278 46 45485 45491 10.1074/jbc.M309069200 12966085

[B41] Armoni M Harel C Karni S Chen H Bar-Yoseph F Ver MR Quon MJ Karnieli E FOXO1 represses peroxisome proliferator-activated receptor-gamma1 and -gamma2 gene promoters in primary adipocytes. A novel paradigm to increase insulin sensitivity J Biol Chem 2006 281 29 19881 19891 10.1074/jbc.M600320200 16670091

[B42] Nielsen R Pedersen TA Hagenbeek D Moulos P Siersbaek R Megens E Denissov S Borgesen M Francoijs KJ Mandrup S Genome-wide profiling of PPARgamma:RXR and RNA polymerase II occupancy reveals temporal activation of distinct metabolic pathways and changes in RXR dimer composition during adipogenesis Genes Dev 2008 22 21 2953 2967 10.1101/gad.501108 18981474 PMC2577787

[B43] Lefterova MI Zhang Y Steger DJ Schupp M Schug J Cristancho A Feng D Zhuo D Stoeckert CJ Jr Liu XS PPARgamma and C/EBP factors orchestrate adipocyte biology via adjacent binding on a genome-wide scale Genes Dev 2008 22 21 2941 2952 10.1101/gad.1709008 18981473 PMC2577797

[B44] Prokesch A Bogner-Strauss JG Hackl H Rieder D Neuhold C Walenta E Krogsdam A Scheideler M Papak C Wong WC Arxes: retrotransposed genes required for adipogenesis Nucleic Acids Res 2010 10.1093/nar/gkq1289 PMC3082915 21177646

[B45] Murholm M Dixen K Qvortrup K Hansen LH Amri EZ Madsen L Barbatelli G Quistorff B Hansen JB Dynamic regulation of genes involved in mitochondrial DNA replication and transcription during mouse brown fat cell differentiation and recruitment PLoS One 2009 4 12 e8458 10.1371/journal.pone.0008458 20107496 PMC2809086

[B46] Seale P Kajimura S Yang W Chin S Rohas LM Uldry M Tavernier G Langin D Spiegelman BM Transcriptional control of brown fat determination by PRDM16 Cell Metab 2007 6 1 38 54 10.1016/j.cmet.2007.06.001 17618855 PMC2564846

[B47] Timmons JA Wennmalm K Larsson O Walden TB Lassmann T Petrovic N Hamilton DL Gimeno RE Wahlestedt C Baar K Myogenic gene expression signature establishes that brown and white adipocytes originate from distinct cell lineages Proc Natl Acad Sci USA 2007 104 11 4401 4406 10.1073/pnas.0610615104 17360536 PMC1810328

[B48] Madsen L Pedersen LM Lillefosse HH Fjaere E Bronstad I Hao Q Petersen RK Hallenborg P Ma T De Matteis R UCP1 induction during recruitment of brown adipocytes in white adipose tissue is dependent on cyclooxygenase activity PLoS One 2010 5 6 e11391 10.1371/journal.pone.0011391 20613988 PMC2894971

[B49] Lukas J Bartkova J Rohde M Strauss M Bartek J Cyclin D1 is dispensable for G1 control in retinoblastoma gene-deficient cells independently of cdk4 activity Mol Cell Biol 1995 15 5 2600 2611 7739541 10.1128/mcb.15.5.2600 PMC230490

[B50] Clarke AR Maandag ER van Roon M van der Lugt NM van der Valk M Hooper ML Berns A te Riele H Requirement for a functional Rb-1 gene in murine development Nature 1992 359 6393 328 330 10.1038/359328a0 1406937

[B51] Pieler R Sanchez-Cabo F Hackl H Thallinger GG Trajanoski Z ArrayNorm: comprehensive normalization and analysis of microarray data Bioinformatics 2004 20 12 1971 1973 10.1093/bioinformatics/bth174 15073026

[B52] Maurer M Molidor R Sturn A Hartler J Hackl H Stocker G Prokesch A Scheideler M Trajanoski Z MARS: microarray analysis, retrieval, and storage system BMC Bioinformatics 2005 6 101 10.1186/1471-2105-6-101 15836795 PMC1090551

[B53] Brazma A Parkinson H Sarkans U Shojatalab M Vilo J Abeygunawardena N Holloway E Kapushesky M Kemmeren P Lara GG ArrayExpress--a public repository for microarray gene expression data at the EBI Nucleic Acids Res 2003 31 1 68 71 10.1093/nar/gkg091 12519949 PMC165538

[B54] Pinent M Hackl H Burkard TR Prokesch A Papak C Scheideler M Hammerle G Zechner R Trajanoski Z Strauss JG Differential transcriptional modulation of biological processes in adipocyte triglyceride lipase and hormone-sensitive lipase-deficient mice Genomics 2008 92 1 26 32 10.1016/j.ygeno.2008.03.010 18572100

[B55] Cheadle C Vawter MP Freed WJ Becker KG Analysis of microarray data using Z score transformation J Mol Diagn 2003 5 2 73 81 10.1016/S1525-1578(10)60455-2 12707371 PMC1907322

[B56] Huang da W Sherman BT Lempicki RA Systematic and integrative analysis of large gene lists using DAVID bioinformatics resources Nat Protoc 2009 4 1 44 57 19131956 10.1038/nprot.2008.211

[B57] Bindea G Mlecnik B Hackl H Charoentong P Tosolini M Kirilovsky A Fridman WH Pages F Trajanoski Z Galon J ClueGO: a Cytoscape plug-in to decipher functionally grouped gene ontology and pathway annotation networks Bioinformatics 2009 25 8 1091 1093 10.1093/bioinformatics/btp101 19237447 PMC2666812

[B58] Sturn A Quackenbush J Trajanoski Z Genesis: cluster analysis of microarray data Bioinformatics 2002 18 1 207 208 10.1093/bioinformatics/18.1.207 11836235

[B59] Subramanian A Tamayo P Mootha VK Mukherjee S Ebert BL Gillette MA Paulovich A Pomeroy SL Golub TR Lander ES Gene set enrichment analysis: a knowledge-based approach for interpreting genome-wide expression profiles Proc Natl Acad Sci USA 2005 102 43 15545 15550 10.1073/pnas.0506580102 16199517 PMC1239896

[B60] Pabinger S Thallinger GG Snajder R Eichhorn H Rader R Trajanoski Z QPCR: Application for real-time PCR data management and analysis BMC Bioinformatics 2009 10 268 10.1186/1471-2105-10-268 19712446 PMC2741456

[B61] Zhao S Fernald RD Comprehensive algorithm for quantitative real-time polymerase chain reaction J Comput Biol 2005 12 8 1047 1064 10.1089/cmb.2005.12.1047 16241897 PMC2716216

[B62] Matys V Kel-Margoulis OV Fricke E Liebich I Land S Barre-Dirrie A Reuter I Chekmenev D Krull M Hornischer K TRANSFAC and its module TRANSCompel: transcriptional gene regulation in eukaryotes Nucleic Acids Res 2006 34 Database D108 110 10.1093/nar/gkj143 PMC1347505 16381825

[B63] Bryne JC Valen E Tang MH Marstrand T Winther O da Piedade I Krogh A Lenhard B Sandelin A JASPAR, the open access database of transcription factor-binding profiles: new content and tools in the 2008 update Nucleic Acids Res 2008 36 Database D102 106 10.1093/nar/gkm955 PMC2238834 18006571

[B64] Quandt K Frech K Karas H Wingender E Werner T MatInd and MatInspector: new fast and versatile tools for detection of consensus matches in nucleotide sequence data Nucleic Acids Res 1995 23 23 4878 4884 10.1093/nar/23.23.4878 8532532 PMC307478

[B65] Benjamini Y Hochberg Y Controlling the False Discovery Rate - a Practical and Powerful Approach to Multiple Testing J Roy Stat Soc B Met 1995 57 1 289 300

