# Supplementary material for: Transcriptional regulatory program in wild-type and retinoblastoma gene-deficient mouse embryonic fibroblasts during adipocyte differentiation
Source: BMC Res Notes. 2011 May 26;4:157. doi: 10.1186/1756-0500-4-157 (PMC3127957; doi:10.1186/1756-0500-4-157)
Supplement: Additional file 2 — Results and primer sequences from q-RT-PCR analysis for validation of microarray experiments and to identify expression levels for specific genes during ME3 and MEFA differentiation. [file 1756-0500-4-157-S2.PDF]

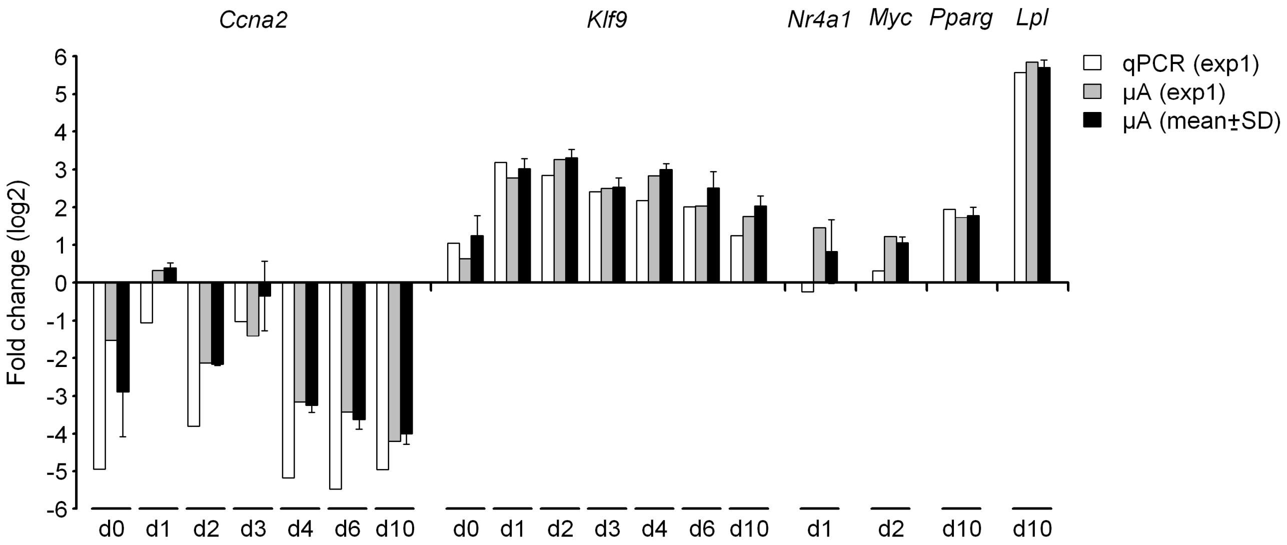

Figure S1: Comparison of microarray data with qPCR data from the first of three experiments for 6 genes at different timepoints during MEF adipocyte differentiation (log2 foldchange between denoted timepoint and preconfluent stage). Additional microarray data averaged over 3 independent experiments (mean±standard deviation) is shown.

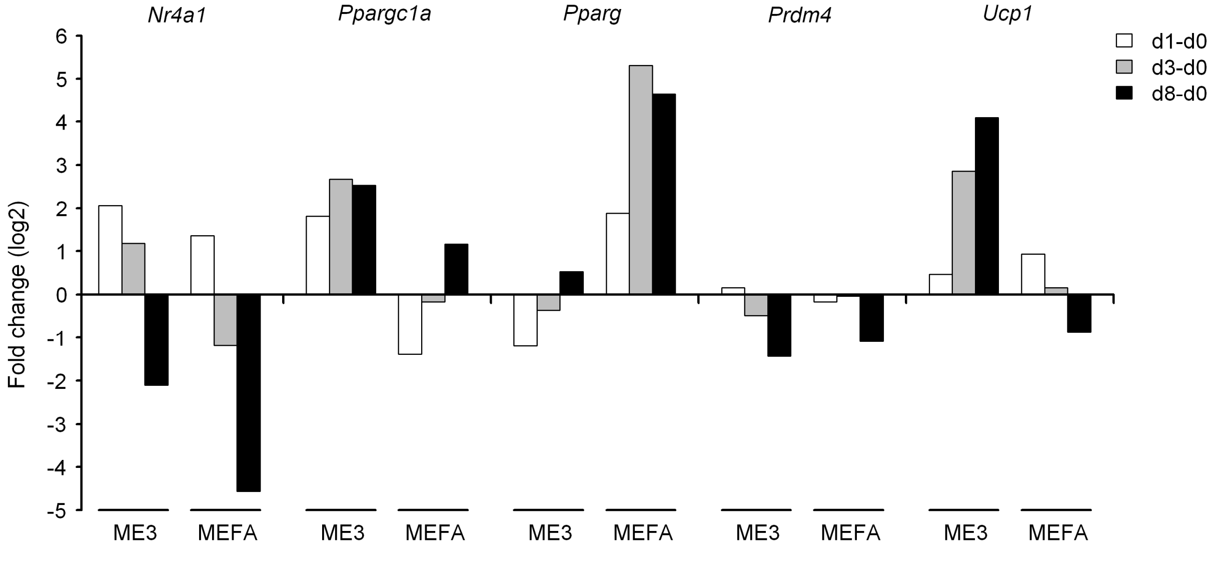

Figure S2: Gene expression data (log2 FC) identified by qPCR analysis for Rb-/-MEFs (ME3) and Rb+/+MEFs (MEFA) cells during differentiation at several timepoints (1 day, 3 days, 8 days) versus undifferentiated cells at timepoint day 0 (start of induction with adipogenic cocktail).

Table S1: Primer sequences for qPCR analysis for validation of microarray data (LUX™ primer, t in the primer sequences indicates where fluorophores are coupled) and to identify expression levels for specific genes during ME3 and MEFA differentiation.

Gene	Forward-Primer	Reverse-Primer	Type/Technology
Pparg	caccaTGCGGAAGCCCTTTGGtG	GGGCGGTCTCCACTGAGAAT	LUX™
Lpl	AGCAGACGCGGGAAGAGATT	caaccaAGGTCTTGCTGCTGTGGTtG	LUX™
Myc	CCCTAGTGCTGCATGAGGAGA	cagcgTTGCTCTTCTTCAGAGTCGCtG	LUX™
Ccna2	CAGAGCTGGCCTGAGTCATTG	gacctaGTGGCGCTTTGAGGTAGGtC	LUX™
Klf9	TGGCTGTGGGAAAGTCTATGG	atacagAAGGGCCGTTCACCTGTAtG	LUX™
Nr4a1	caaccaAGGTCTTGCTGCTGTGGTtG	GGCTTCTTCAAGCGCACAGT	LUX™
Ucp1	ACACCTGCCTCTCTCGGAAA	TAGGCTGCCCAATGAACACT	SYBR® green
Ppargc1a	CCACACCCACAGGATCAGAA	TCTTCGCTTTATTGCTCCATGA	SYBR® green
Pparg2	TGCCTATGAGCACTTCACAAGAAAT	CGAAGTTGGTGGGCCAGAA	SYBR® green
Nr4a1	GCATACCGATCTAAACCCGGT	CGTGTCGATCAGTGATGAGGA	SYBR® green
Prdm4	GAGACCGAGGAGTGATGTGTG	TGCCAGGTAGAATGGTTCTGAA	SYBR® green
Uxt	CTCACAGAGCTCAGCGACAGC	AAATTCTGCAGGCCTTGTAGTTCTC	SYBR® green
